# Mechanism of foreign DNA recognition by a CRISPR RNA-guided surveillance complex from *Pseudomonas aeruginosa*

**DOI:** 10.1093/nar/gkv094

**Published:** 2015-02-08

**Authors:** MaryClare F. Rollins, Jason T. Schuman, Kirra Paulus, Habib S.T. Bukhari, Blake Wiedenheft

**Affiliations:** 1 Department of Microbiology and Immunology, Montana State University, Bozeman, MT 59717, USA; 2 GE Life Sciences, Piscataway, NJ 08854, USA; 3 Biozentrum, University of Basel, Klingelbergstrasse 50/70, 4056 Basel, Switzerland

## Abstract

The Type I-F CRISPR-mediated (clustered regularly interspaced short palindromic repeats) adaptive immune system in *Pseudomonas aeruginosa* consists of two CRISPR loci and six CRISPR-associated (*cas*) genes. Foreign DNA surveillance is performed by a complex of Cas proteins (Csy1–4) that assemble with a CRISPR RNA (crRNA) into a 350-kDa ribonucleoprotein called the Csy complex. Here, we show that foreign nucleic acid recognition by the Csy complex proceeds through sequential steps, initiated by detection of two consecutive guanine–cytosine base pairs (G–C/G–C) located adjacent to the complementary DNA target. We show that this motif, called the PAM (protospacer adjacent motif), must be double-stranded and that single-stranded PAMs do not provide significant discriminating power. Binding assays performed with G–C/G–C-rich competitor sequences indicate that the Csy complex interacts directly with this dinucleotide motif, and kinetic analyses reveal that recognition of a G–C/G–C motif is a prerequisite for crRNA-guided binding to a target sequence. Together, these data indicate that the Csy complex first interacts with G–C/G–C base pairs and then samples adjacent target sequences for complementarity to the crRNA guide.

## INTRODUCTION

Bacteria and archaea have evolved sophisticated nucleic acid-based adaptive immune systems to defend against exogenous genetic elements like viruses and plasmids (1–6). Immunity is acquired by integrating short segments of foreign DNA into one end of the host encoded CRISPR locus (clustered regularly interspaced short palindromic repeats). These foreign sequences, called spacers, are flanked by short repeat sequences, creating the repeat–spacer–repeat pattern that is characteristic of CRISPR-mediated immune systems. CRISPR loci are transcribed and processed into short CRISPR RNAs (crRNAs), which are incorporated into large ribonucleoprotein complexes that scan the intracellular environment for foreign nucleic acid sequences complementary to the crRNA spacer. Hybridization between a crRNA spacer sequence and a complementary foreign target sequence, called a protospacer (i.e. origin of the spacer), triggers degradation of the invasive DNA or RNA by CRISPR-associated (Cas) nucleases.

CRISPR–Cas systems are widespread and phylogenetically diverse. Three major types (Types I, II and III) have been described, comprising at least 11 subtypes (IA-F, IIA-C and IIIA-B) that encode distinct crRNA-guided surveillance complexes (1,5,7). While some Type III systems target RNA (8–12), the Types I and II systems target and destroy invading DNA (13–19). All of these crRNA-guided surveillance complexes must locate target sequences on a time scale that affords protection from rapidly replicating phages, and CRISPR systems that target DNA must also be able to reliably distinguish complementary spacer sequences in the host CRISPR locus (self) from identical protospacer sequences in the invading DNA target (non-self). In Types I and II systems, this distinction is accomplished by recognition of a short (2–4 base pairs) sequence called a protospacer adjacent motif (PAM) (20–23). The PAM is only found next to complementary protospacer targets in foreign DNA, and is absent from repeat sequences that flank complementary spacer sequences in the host CRISPR locus.

Recent structural and biochemical studies of the crRNA-guided Cas9 protein from *Streptococcus pyogenes* (Type II) have revealed that Cas9 recognizes a 5′-NGG-3′ PAM through major and minor groove interactions, and mutations that disrupt the GG result in substantial binding defects (18,24–25). This mechanism of PAM recognition is distinct from PAM detection by the crRNA-guided surveillance complex from *Escherichia coli* (Type IE), which recognizes up to 27 different variation of a 3-nt PAM (23), and mutations in the non-target strand PAM do not interfere with target recognition (17,26) (Supplemental Figure S1).

Here, we show that target recognition by the foreign DNA surveillance complex from *Pseudomonas aeruginosa* (Type I-F) initiates with PAM recognition and then proceeds by crRNA-guided base pairing to target DNA. Kinetic analyses suggest an order of operations for target binding, in which the complex first associates with double-stranded DNA (dsDNA) through non-sequence-specific interactions characterized by rapid on- and off-rates. These non-specific interactions are stabilized by interactions with the PAM, which are required prior to sampling of adjacent sequences for potential hybridization with the crRNA guide. Our data provide direct evidence for double-stranded PAM recognition by the Csy complex, and suggest a central role for the PAM in rapid surveillance and detection of invading nucleic acid targets.

## MATERIALS AND METHODS

### Expression and purification of the Csy complex

*Csy* genes and a synthetic crRNA were co-expressed on separate vectors in *E. coli* BL21 (DE3) cells as previously described (27). Expression was induced with 0.5 mM isopropyl-β-d-1-thiogalactopyranoside (IPTG) at OD_600_ = 0.5 nm. Cells were incubated overnight at 16°C, then pelleted by centrifugation (5000 x *g* for 15 min at 4°C) and re-suspended in lysis buffer (50 mM 4-(2-hydroxyethyl)-1-piperazineethanesulfonic acid (HEPES) pH7.5, 300 mM potassium chloride, 5% glycerol, 1 mM Tris(2-carboxyethyl) phosphine hydrochloride (TCEP), 1x protease inhibitor cocktail (Thermo Scientific)). Pellets were sonicated on ice for 2 × 2.5 min (1 s on, 3 s off), then lysate was clarified by centrifugation at 22 000 x *g* for 30 min at 4°C. The Csy complex self-assembles *in vivo* and the intact complex was affinity purified on Ni-NTA resin (Qiagen) using hexa-histidine tags on either Csy3 or Csy1. Elution was performed using the lysis buffer supplemented with 300 mM imidazole. Tobacco etch virus (TEV) protease was added to remove the N-terminal His_6_ tags and the sample was dialyzed at 4°C overnight in gel filtration buffer (20 mM HEPES pH 7.5, 100 mM KCl, 5% glycerol, 1 mM TCEP). Protein was concentrated (Corning Spin-X concentrators) at 4°C before further purification on a Superdex 200 size-exclusion column (GE Healthcare).

### Electrophoretic mobility shift assays (EMSA)

Binding assays were performed by incubating a concentration gradient (0, 0.001, 0.01, 0.05, 0.1, 0.5, 1, 10, 100, 1000 nM) of Csy complex with <0.5 nM of 5′ ^32^P-labeled DNA oligonucleotides for 15 minutes at 37°C in reaction buffer (20 mM HEPES pH 7.5, 100 mM KCl, 5% glycerol, 1 mM TCEP, 2 mM Ethylenediaminetetraacetic acid (EDTA)). Reaction products were run on 6% polyacrylamide gels, which were dried and imaged with a phosphor storage screen (Kodak), then scanned with a Typhoon phosphorimager (GE Healthcare). Bands were quantified using ImageQuant software, and the percent DNA bound was plotted as a function of Csy complex concentration, then fit with a standard binding isotherm:

\begin{eqnarray*} &&{\rm Fraction}\,{\rm DNA}\,{\rm bound} = \nonumber \\ &&[{\rm Csy}\,{\rm complex}]/({K}_{\rm D} + [{\rm Csy}\,{\rm complex}]) \end{eqnarray*}

Reported *K*_D_s represent the average value from three independent experiments. Competition assays were quantified at 100 nM Csy complex at 37°C for 5 min, using 0.66 μM competitor DNA.

### Surface plasmon resonance (SPR)

Experiments were conducted with a Biacore X100 SPR instrument (GE Healthcare). 5′-Biotinylated DNA oligonucleotides were immobilized on the surface of a streptavidin-coated sensor chip (GE Healthcare). Purified Csy complex was injected into the flow cell, and Csy complex–DNA binding events were recorded in real time. Experiments were conducted at 37°C, in 20 mM HEPES pH 7.5, 100 mM KCl, 1 mM TCEP, 0.02% Tween, 50 μM EDTA. Data were analyzed using Biacore X100 evaluation software. Sensorgrams for non-target DNA and target DNAs with T–A/T–A PAM were fit with a Langmuir binding model. Sensorgrams for target DNA with G–C/G–C PAM were fit with a Langmuir model for the dissociation phase only, in order to determine the *k*_d_. The half-life of the dissociation was calculated as ln 2/*k*_d_. Sensorgrams for target DNA with G–C/G–C PAM were also fit using a two-state model to facilitate qualitative analysis of this interaction.

## RESULTS

### PAM recognition

**Figure 1.**
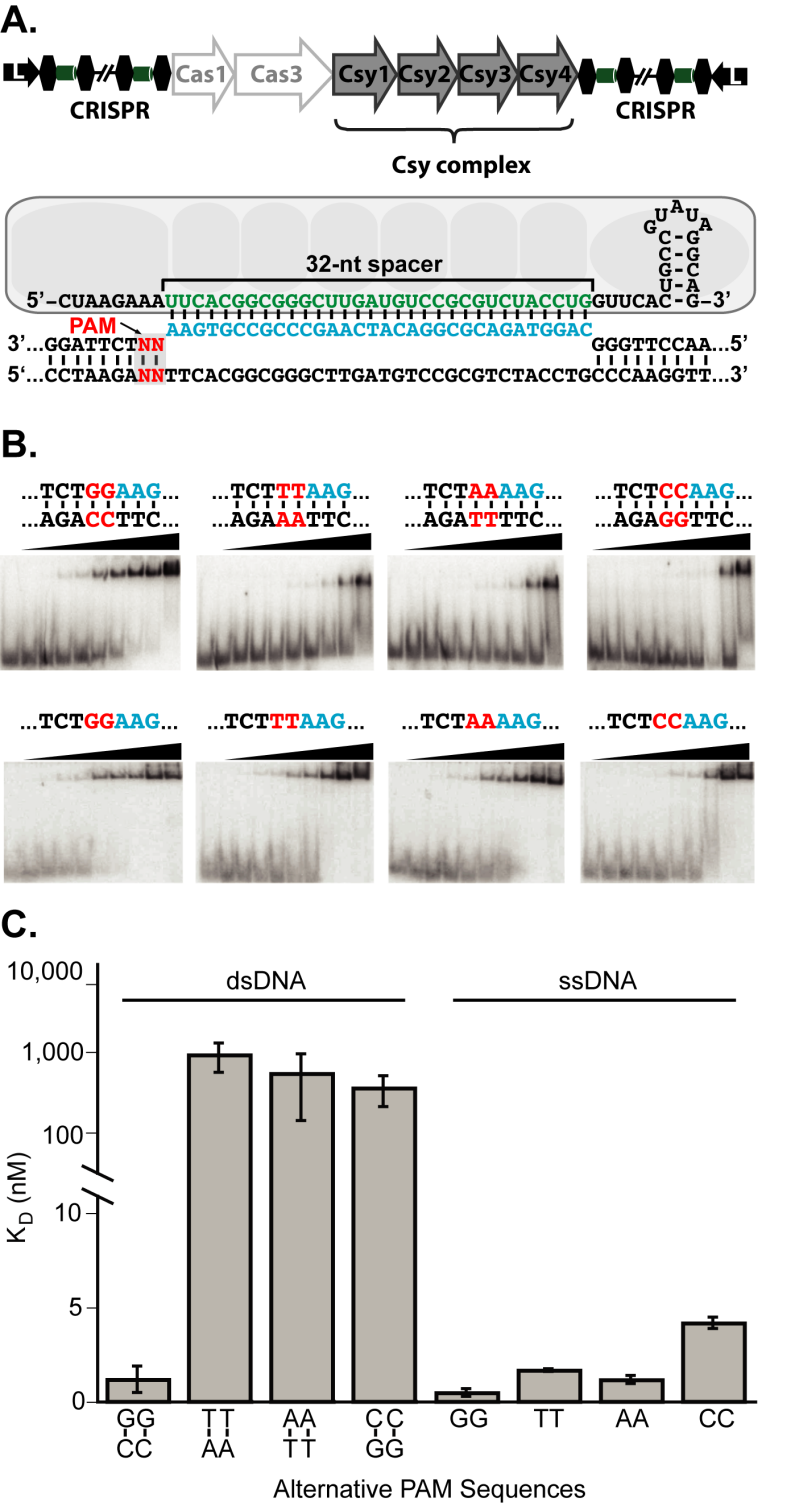
The Csy complex recognizes a double-stranded GC/GC PAM. (**A**) Organization of the CRISPR-Cas locus in the *P. aeruginosa* (PA14) genome and a schematic representation of the Csy complex. Csy protein subunits (light gray ovals) assemble with a crRNA, which includes a 32-nt spacer sequence (green). The Csy complex binds target DNA through direct hybridization between the crRNA spacer and a complementary target sequence (protospacer) (blue). The PAM (red) immediately 3′ of the protospacer is critical for crRNA-guide stand invasion in double-stranded DNA targets. (**B**) EMSAs performed with increasing concentrations of Csy complex (see ‘Materials and Methods' section) incubated with ssDNA or dsDNA 80-nt targets containing fully complementary protospacers and alternate PAM sequences (shown in red). Binding was quantified and plotted as a function of protein concentration, then fit with binding isotherms to determine equilibrium dissociation constants (*K*_D_) (see ‘Materials and Methods' section). (**C**) Binding affinities for Csy complex with dsDNA and ssDNA targets with alternate PAMs. Non-GG PAMs severely abrogated binding to dsDNA targets, but had comparably minor effects in ssDNA targets.

*Pseudomonas aeruginosa* is an environmentally ubiquitous gram-negative bacterium and an opportunistic human pathogen (28). The genome of *P. aeruginosa* (strain PA14) contains an active Type IF CRISPR-Cas system (29–31), which includes 6 *cas* genes (*cas1, cas3, csy1, csy2, csy3* and *csy4*), and two CRISPR loci (Figure 1A). The Csy proteins assemble into a stable ribonucleoprotein complex consisting of one Csy1, one Csy2, six Csy3, one Csy4 and one crRNA (27). This complex, referred to as the Csy complex, engages DNA targets through sequence-specific hybridization with the crRNA guide. Phage challenge experiments in PA14 indicate that new spacers are acquired from sequences in the phage genome with GG PAMs (5′-protospacer,GG-3′), and the presence of this PAM is critical for protection (29,30).

To investigate the importance of the PAM in crRNA-guided target binding by the Csy complex, we performed native gel mobility shift assays using a series of double-stranded DNA (dsDNA) and single-stranded DNA (ssDNA) substrates containing protospacer sequences flanked by one of four different dinucleotides (Figure 1A and B, Supplemental Tables S1 and S2). Double- and single-stranded DNA substrates containing a protospacer and a G–C/G–C or a GG PAM respectively; bound with high affinity (dsDNA *K*_D_ ∼1 nM; ssDNA *K*_D_ ∼0.5 nM). Substitution of the GG with alternative dinucleotides resulted in binding defects for both ssDNA and dsDNA substrates; however, there was a pronounced difference in the magnitude of the effect between dsDNA and ssDNA (Figure 1B). The largest difference in binding affinity for ssDNA substrates was 8-fold, with an average difference closer to 2-fold. In contrast, the same substitutions made in double-stranded targets resulted in 100-fold weaker binding affinities. These data indicate that the Csy complex distinguishes between PAMs in a double-stranded context, and that the presence of a G–C/G–C PAM is required for high-affinity binding of dsDNA targets.

### PAM is double-stranded

**Figure 2.**
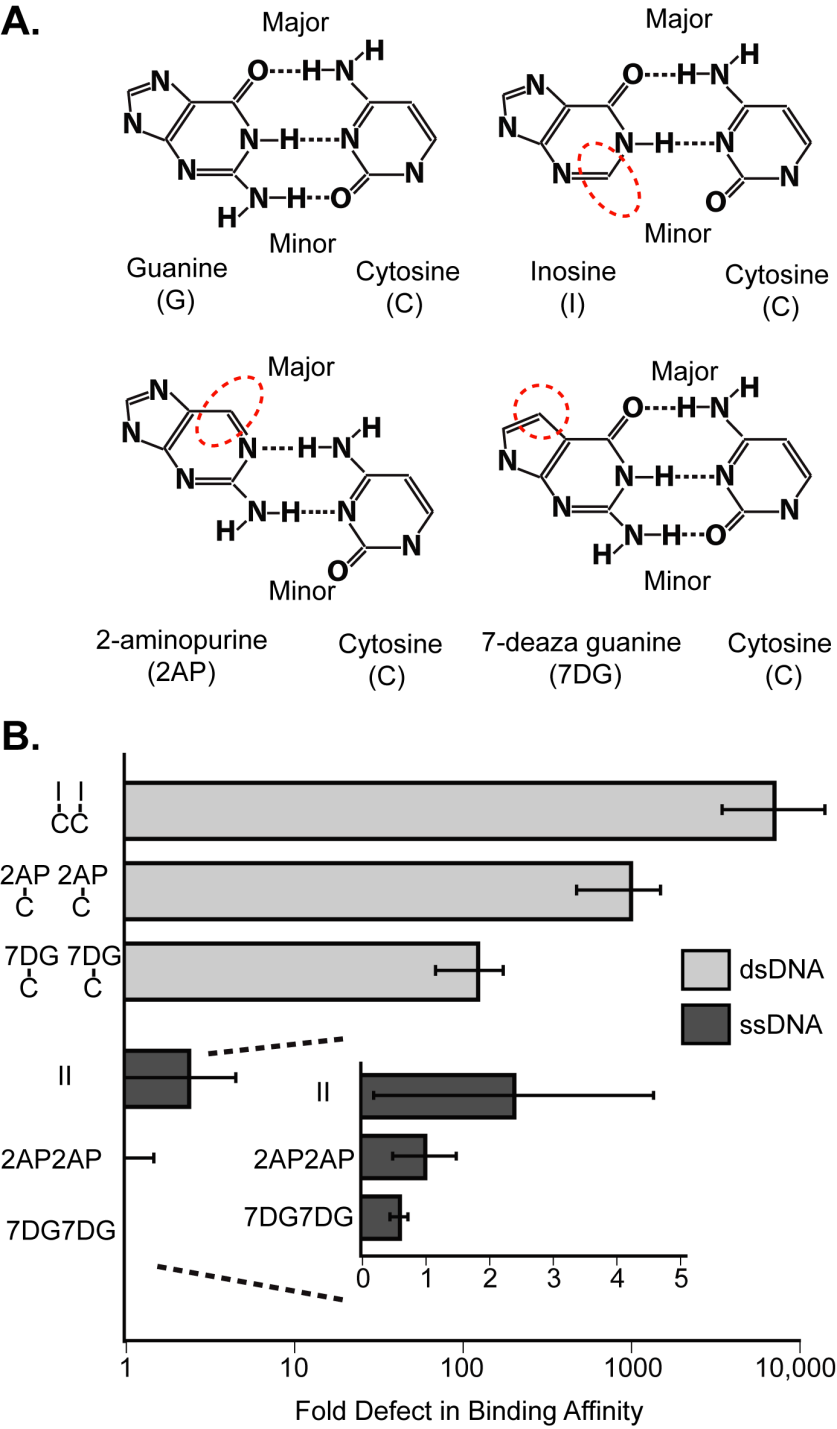
Changes to the chemical signature of a double-stranded PAM result in binding defects. (**A**) DNA targets were generated using purine analogs in place of the two guanines in the canonical G–C/G–C PAM. The nucleotide analogs present altered chemical signatures in either the major (2AP or 7DG) or minor groove (inosine) of the DNA duplex. Dashed red ovals highlight missing functional groups. (**B**) Binding affinities for dsDNA and ssDNA targets with chemically modified PAMs were tested by EMSAs. Targets containing modified PAMs severely abrogated binding in dsDNA targets, but had no significant effect in ssDNA. Binding defects were quantified as the fold increase in *K*_D_ (nM) relative to a G–C/G–C PAM (for dsDNA) or GG PAM (for ssDNA).

Since PAM discrimination is significantly more stringent in double-stranded DNA targets, we hypothesized that PAM recognition relies on chemical signatures presented in the major or minor groove of the PAM duplex. To test this hypothesis, we repeated the band shift assays using dsDNA substrates in which the guanines of the PAM were replaced by purine analogs lacking specific functional groups (Figure 2A, and Supplemental Table S2). Changes to the chemical presentation of the PAM in either the major or minor groove resulted in attenuated binding affinities, ranging from ∼120-fold weaker binding for the 7-deaza guanine PAM (major groove), to >1000-fold weaker binding for the inosine PAM (minor groove) (Figure 2B and Supplemental Figure S2). In contrast, ssDNA targets with the same PAM modifications showed no significant reduction in binding affinities. These results suggest that chemical signatures in both the major and minor groove contribute to PAM recognition by the Csy complex.

### Kinetics of target recognition

**Figure 3.**
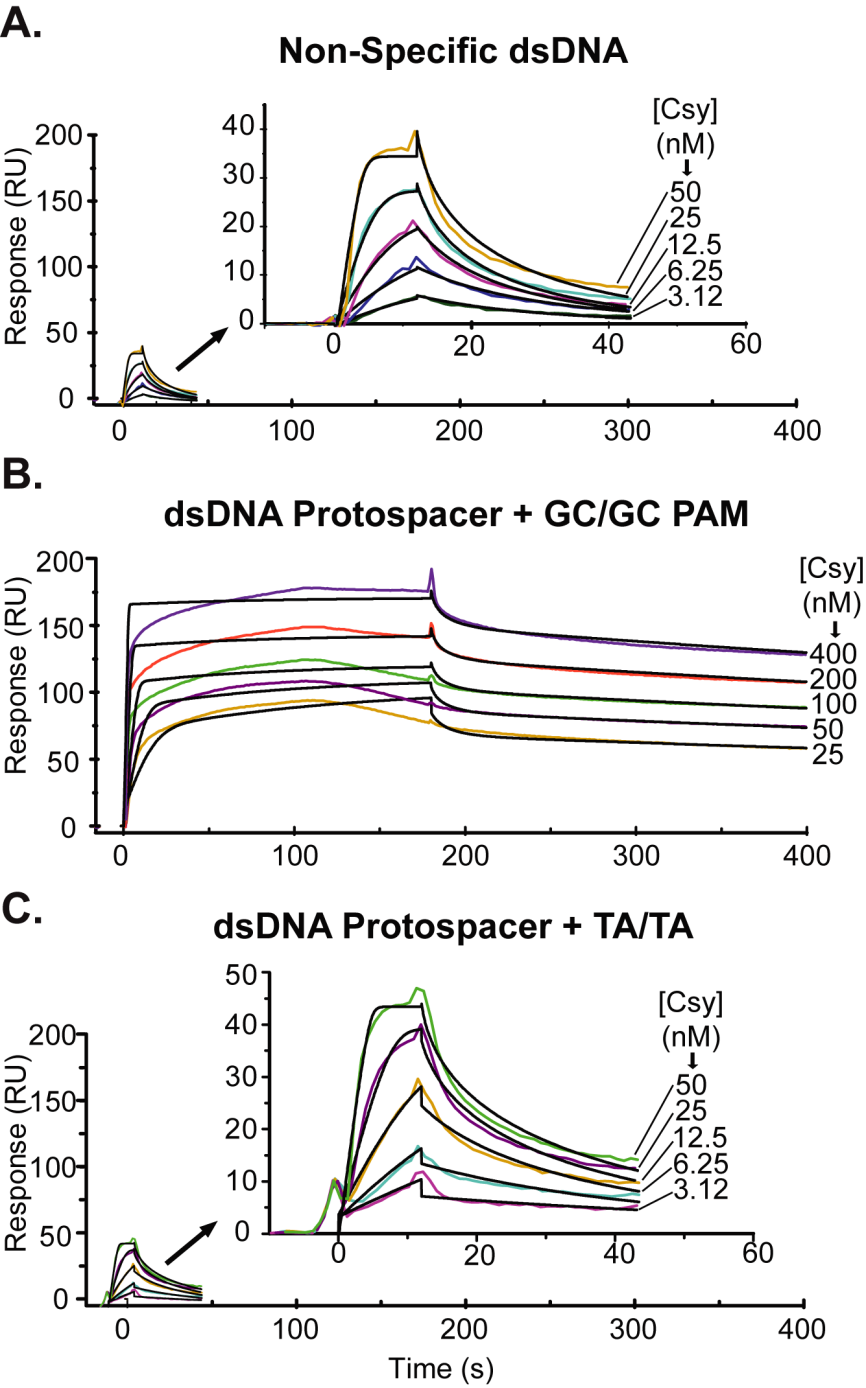
Recognition of two consecutive GC base pairs is a prerequisite for target binding. Binding kinetics of the Csy complex were analyzed using surface plasmon resonance (SPR). Kinetic data for a concentration series of Csy complex (colored lines) were fit using either a Langmuir or two state-binding model (black lines). (**A**) Experiments performed using non-target dsDNA (no protospacer) revealed non-specific binding characterized by rapid on- and off-rates (*k*_a_ = 1.1 × 10^7^ M^−1^ s^−1^; *k*_d_ = 1.3 × 10 s^−1^). (**B**) dsDNA targets containing a full protospacer and G–C/G–C PAM were bound with high affinity. Kinetic data were fit using a two-state binding model for a qualitative analysis. (**C**) Binding kinetics for dsDNA targets containing full protospacers flanked by a mutated PAM (i.e. T–A/T–A) are indistinguishable from binding kinetics for non-target dsDNA (*k*_a_ = 1.9 × 10^7^ M^−1^ s^−1^; *k*_d_ = 1.1 × 10 s^−1^).

CRISPR RNA-guided surveillance complexes must be able to efficiently locate foreign targets in a crowded intracellular environment that contains megabases of non-target DNA. We hypothesize that the Csy complex accelerates target location through complex binding behaviors that involve fast association- and dissociation-rates with non-target sequences, and that these transient interactions are stabilized by protein-mediated recognition of the PAM. To test these hypotheses, we used surface plasmon resonance (SPR) to measure real-time binding kinetics of the Csy complex to a series of dsDNA substrates (Figure 3 and Supplemental Table S2). First, we measured the association- and dissociation-rates of the Csy complex with dsDNA containing neither a protospacer nor a PAM. These interactions are extremely fast (*k*_a_ = 1.1 × 10^7^ M^−1^ s^−1^; *k*_d_ = 1.3 × 10^−1^ s^−1^; *τ*_1/2_ = 5.3 s) (Figure 3A) and are sensitive to ionic strength (data not shown), consistent with an electrostatic interaction.

Next, we tested binding of the Csy complex to a dsDNA target containing a protospacer and a G–C/G–C PAM (Figure 3B). The kinetic data for this interaction cannot be fit using a Langmuir binding model (Supplemental Figure S3A), which is consistent with a complex interaction involving more than one step. To account for the fast interactions measured for non-sequence specific DNA, and for the longer-lived associations with the target sequence, we fit the data using a two-state binding model (Figure 3B and Supplemental Figure S3B). The two-state model has been used to describe the binding kinetics of sequence-specific DNA-binding proteins (32–34), which have been shown to locate their target sequences using distinct ‘search’ and ‘recognition’ modes (35). While the two-state binding model exaggerates the association rate of the Csy complex, the dissociation phase of this data is modeled with high confidence, suggesting that interactions with authentic targets containing both a protospacer and a PAM are extremely stable. To compare the stability of Csy complex with non-specific dsDNA or a dsDNA target containing a PAM and a protospacer, we fit only the stable dissociation data (10 s after the start of dissociation) with a Langmuir model. Analyzing the dissociation phase independently simplifies the fitting, and allows quantification of binding stability. The resulting *k*_d_ (6.5 × 10^−4^ s^−1^) indicates the half-life (*τ*_1/2_ = 1066 s) of the Csy complex bound to a target sequence with a G–C/G–C PAM is dramatically (>150×) longer (more stable) than for non-target sequences (Figure 3A), or targets with incorrect PAMs (Figure 3C).

To determine the role of the PAM in target recognition, we measured the kinetics of Csy complex binding dsDNA oligonucleotides containing an identical protospacer sequence flanked by a T–A/T–A dinucleotide in place of the G–C/G–C PAM (Figure 3C). Despite the presence of a complementary protospacer, the binding behavior for this target is indistinguishable from non-target dsDNA containing neither a protospacer nor PAM (*k*_a_ = 1.9 × 10^7^ M^−1^ s^−1^; *k*_d_ = 1.1 × 10 s^−1^; *τ*_1/2_ = 6.3 s). Similar to what was observed for non-specific dsDNA (Figure 3A), the kinetic rate constants for this interaction result in a *K*_D_ (6 nM) that is more than two orders of magnitude lower than the equilibrium dissociation constant estimated from gel shift assays. However, this is not unexpected since EMSAs measure the stability of bound products, and this method has been shown to substantially overestimate *K*_D_s for interactions with very fast dissociations (36). Regardless of the absolute affinities, kinetic data from dsDNA substrates containing a protospacer flanked by a T–A/T–A are identical to non-specific DNA, suggesting that recognition of a double-stranded PAM must occur before the Csy complex can recognize a protospacer.

### PAM scanning by the Csy complex

If the Csy complex scans dsDNA for sequential G–C base pairs, we would expect this to be a relatively weak interaction. Strong binding to G–C/G–C base pairs would increase residence time on a sequence motif that occurs randomly every 16 base pairs, collectively slowing the overall target search and compromising the CRISPR immune response. In contrast, weaker interactions that momentarily stabilize the complex at G–C/G–C pairs might allow for crRNA-guided sampling of the adjacent DNA.

**Figure 4.**
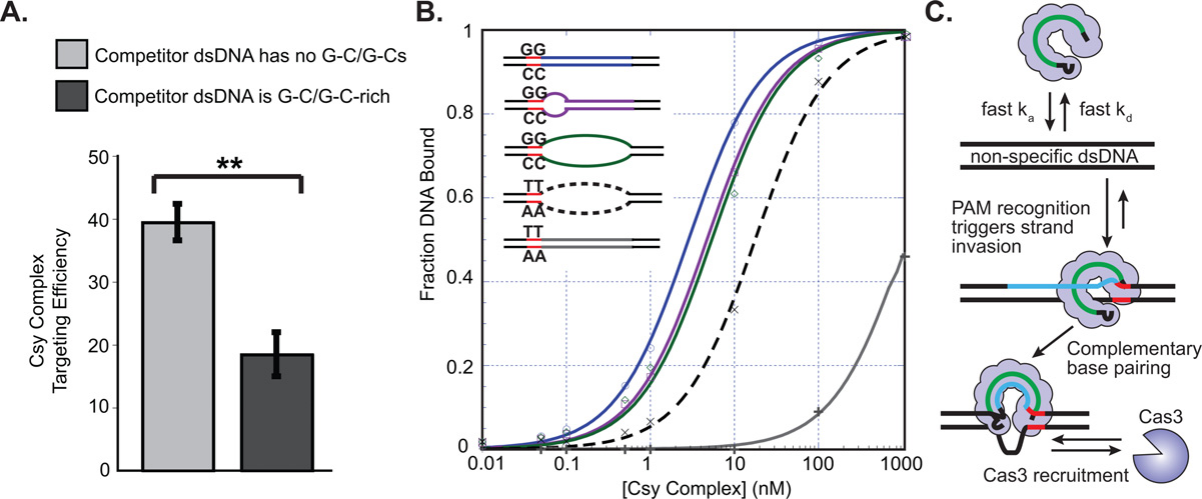
PAM binding promotes strand invasion. (**A**) EMSAs were performed by incubating [^32^P]-radiolabeled dsDNA targets with unlabeled dsDNA competitors containing either no G–C/G–C pairs, or 13 G–C/G–C base pairs (G–C/G–C-rich). The presence of G–C/G–C-rich competitor DNA significantly reduced the amount of labeled target bound by the Csy complex. Targeting efficiency for the Csy complex in the presence of both competitors was calculated as a percentage of complete target binding (i.e. total target binding in the absence of competitor DNA). (**B**) dsDNA targets were designed with bubbles in the seed sequence, or the entire protospacer. Pre-melted protospacer sequences (purple or green) flanked by a G–C/G–C PAM were not bound with greater affinity than fully duplexed targets with G–C/G–C PAMs (blue). However, a protospacer bubble did increase the binding affinities for targets with a T–A/T–A PAM (dashed black line versus gray). (**C**) Model of the Csy complex target search process. The Csy complex engages in rapid, non-sequence-specific interactions with dsDNA. Encounters with G–C/G–C dinucleotides provide additional weak but specific interactions that facilitate ATP-independent strand invasion. Complementary base pairing between the crRNA spacer and the protospacer DNA target results in a stable interaction that recruits Cas3 for degradation of the target DNA.

To test this ‘PAM scan’ model, we performed gel shift assays using labeled dsDNA targets and unlabeled dsDNA competitors containing either no GC/GC base pairs, or 13 GC/GC base pairs (Figure 4A and Supplemental Table S2). We anticipated that GC/GC-rich competitor DNA would sequester the Csy complex more effectively than competitor DNA with no GC/GCs, resulting in reduced binding of the labeled target. Accordingly, in reactions containing a high molar excess of the GC/GC-rich competitor, we detected a significant reduction in target DNA binding efficiency (Figure 4A and Supplemental Figure S4). These data suggest that recognition of the G–C/G–C base pair transiently stabilizes the interaction between the Csy complex and dsDNA.

### Functional importance of the PAM

In the Type II CRISPR system in *S. pyogenes*, PAM recognition by Cas9 has been shown to destabilize dsDNA, thus facilitating strand invasion (25). To test for a similar mechanism in the Csy system, we created a series of dsDNA targets containing identical target strand protospacers, but the non-complementary DNA strand was designed with mismatches (Figure 4B). We hypothesized that pre-melting the protospacer duplex would increase accessibility to the target strand sequences, resulting in higher binding affinities. However, binding affinities for DNA targets with a bubble in the first eight nucleotides of the protospacer (i.e. seed region) or a bubble across entire protospacer were no stronger than binding affinities for a completely duplexed target (Figure 4B). Thus, pre-melting the protospacer does not improve target binding for substrates that contain a PAM. However, for substrates not flanked by a PAM (e.g. TA/TA), pre-melting the protospacer sequence dramatically improves target binding as compared to duplexed sequences (Figure 4B). Collectively, these data suggest that the primary role of PAM recognition by the Csy complex is to facilitate crRNA-guided strand invasion by destabilizing the target duplex.

## DISCUSSION

To provide effective defense, crRNA-guided surveillance systems must locate target sequences efficiently within a crowded cellular environment, while reliably avoiding complementary sequences in the bacterial genome. Here we show that the Csy complex from *P. aeruginosa* targets foreign DNA through complex mechanisms involving protein-mediated interactions with DNA and crRNA-guided interactions with the complementary DNA (Figure 4C). The target search is initiated by rapid association and dissociation rates with non-target double-stranded DNA. Encounters with two sequential G–C base pairs provide weak, but specific interactions that are necessary for crRNA-guided strand invasion. Hybridization with the target DNA proceeds along the length of the crRNA spacer, and may induce a conformational change similar to what has been observed for the Cascade complex from *E. coli* (14,37–40). In all Type I systems, the target bound surveillance complex serves as a molecular beacon that recruits a trans-acting nucleases called Cas3 (Figure 4C) (1,7,37).

Anders *et al*. recently reported the crystal structure of the CRISPR-associated protein Cas9 from *S. pyogenes* in complex with a small guide RNA and bound to a DNA target with a double-stranded PAM (25). The PAM in this system consists of a 5′-NGG-3′ motif where the two guanosines are located on the displaced strand. The structure reveals major and minor groove interactions where the guanidinium groups of two arginines reach into the major groove and form base specific hydrogen bonds with the two guanosines. While the residues involved in PAM recognition by the Csy complex awaits high-resolution structural data, it is interesting to note that chemical modifications to the edges of the guanosines presented in either the major or minor groove of the double-stranded PAM destabilize target binding (Figure 2).

The Csy complex appears to use a ‘PAM scan’ mechanism for target location, similar to Cas9. Sternberg *et al*. demonstrated that Cas9 locates target sequences by first interacting with PAMs, which allows sampling of the adjacent sequence (24). The authors propose that this may be the predominant mechanism for target search in CRISPR effector complexes. Our work with the Csy complex supports this suggestion. However, PAM recognition by the crRNA-guided surveillance complex from *Streptococcus thermophilus* is promiscuous, requiring only a single A–T base pair (41), and the Cascade complex from *E. coli* (Type I-E) recognizes protospacers flanked by 27 different variants of a 3-nt PAM (23) (Supplemental Figure S1). Five of the PAM motifs recognized by Cascade elicit degradation of the target via Cas3 recruitment, and another 22 different variants results in rapid integration of new spacer sequences derived from the target template (23). These data suggest that PAM recognition by Cascade is promiscuous and may point toward a PAM-independent mechanism for protospacer recognition. The difference in this fundamental process between the Type I-E system and its nearest phylogenetic neighbor, the Type IF system, may signal unanticipated mechanistic diversity across all CRISPR system subtypes.

## SUPPLEMENTARY DATA

Supplementary Data are available at NAR Online.

SUPPLEMENTARY DATA

## References

[1] Sorek R., Lawrence C.M., Wiedenheft B. (2013). CRISPR-mediated adaptive immune systems in bacteria and archaea. Annu. Rev. Biochem..

[2] Barrangou R., Marraffini L.A. (2014). CRISPR–Cas systems: prokaryotes upgrade to adaptive immunity. Mol. Cell.

[3] Wiedenheft B., Sternberg S.H., Doudna J.A. (2012). RNA-guided genetic silencing systems in bacteria and archaea. Nature.

[4] Bondy-Denomy J., Davidson A.R. (2014). To acquire or resist: the complex biological effects of CRISPR-–Cas systems. Trends Microbiol..

[5] van der Oost J., Westra E.R., Jackson R.N., Wiedenheft B. (2014). Unravelling the structural and mechanistic basis of CRISPR–Cas systems. Nat. Rev. Microbiol..

[6] Heler R., Marraffini L.A., Bikard D. (2014). Adapting to new threats: the generation of memory by CRISPR–Cas immune systems. Mol. Microbiol..

[7] Makarova K.S., Haft D.H., Barrangou R., Brouns S.J., Charpentier E., Horvath P., Moineau S., Mojica F.J., Wolf Y.I., Yakunin A.F. (2011). Evolution and classification of the CRISPR–Cas systems. Nat. Rev. Microbiol..

[8] Hale C.R., Zhao P., Olson S., Duff M.O., Graveley B.R., Wells L., Terns R.M., Terns M.P. (2009). RNA-guided RNA cleavage by a CRISPR RNA–Cas protein complex. Cell.

[9] Staals R.H., Agari Y., Maki-Yonekura S., Zhu Y., Taylor D.W., van Duijn E., Barendregt A., Vlot M., Koehorst J.J., Sakamoto K. (2013). Structure and activity of the RNA-targeting Type III-B CRISPR–Cas complex of Thermus thermophilus. Mol. Cell.

[10] Hale C.R., Cocozaki A., Li H., Terns R.M., Terns M.P. (2014). Target RNA capture and cleavage by the Cmr type III-B CRISPR–Cas effector complex. Genes Dev..

[11] Tamulaitis G., Kazlauskiene M., Manakova E., Venclovas C., Nwokeoji A.O., Dickman M.J., Horvath P., Siksnys V. (2014). Programmable RNA shredding by the Type III-A CRISPR–Cas system of Streptococcus thermophilus. Mol. Cell.

[12] Benda C., Ebert J., Scheltema R.A., Schiller H.B., Baumgärtner M., Bonneau F., Mann M., Conti E. (2014). Structural model of a CRISPR RNA-silencing complex reveals the RNA-target cleavage activity in Cmr4. Mol. cell.

[13] Garneau J.E., Dupuis M.E., Villion M., Romero D.A., Barrangou R., Boyaval P., Fremaux C., Horvath P., Magadan A.H., Moineau S. (2010). The CRISPR/Cas bacterial immune system cleaves bacteriophage and plasmid DNA. Nature.

[14] Jore M.M., Lundgren M., van Duijn E., Bultema J.B., Westra E.R., Waghmare S.P., Wiedenheft B., Pul U., Wurm R., Wagner R. (2011). Structural basis for CRISPR RNA-guided DNA recognition by Cascade. Nat. Struct. Mol. Biol..

[15] Sinkunas T., Gasiunas G., Fremaux C., Barrangou R., Horvath P., Siksnys V. (2011). Cas3 is a single-stranded DNA nuclease and ATP-dependent helicase in the CRISPR/Cas immune system. EMBO J..

[16] Mulepati S., Bailey S. (2013). In vitro reconstitution of an Escherichia coli RNA-guided immune system reveals unidirectional, ATP-dependent degradation of DNA target. J. Biol. Chem..

[17] Westra E.R., van Erp P.B.G., Künne T., Wong S.P., Staals R.H.J., Seegers C.L.C., Bollen S., Jore M.M., Semenova E., Severinov K. (2012). CRISPR immunity relies on the consecutive binding and degradation of negatively supercoiled invader DNA by Cascade and Cas3. Mol. Cell.

[18] Jinek M., Chylinski K., Fonfara I., Hauer M., Doudna J.A., Charpentier E. (2012). A programmable dual-RNA-guided DNA endonuclease in adaptive bacterial immunity. Science.

[19] Gasiunas G., Barrangou R., Horvath P., Siksnys V. (2012). Cas9-crRNA ribonucleoprotein complex mediates specific DNA cleavage for adaptive immunity in bacteria. Proc. Natl. Acad. Sci. U.S. A..

[20] Deveau H., Barrangou R., Garneau J.E., Labonte J., Fremaux C., Boyaval P., Romero D.A., Horvath P., Moineau S. (2008). Phage response to CRISPR-encoded resistance in Streptococcus thermophilus. J. Bacteriol..

[21] Horvath P., Coute-Monvoisin A.C., Romero D.A., Boyaval P., Fremaux C., Barrangou R. (2008). Comparative analysis of CRISPR loci in lactic acid bacteria genomes. Int. J. Food Microbiol..

[22] Mojica F.J., Diez-Villasenor C., Garcia-Martinez J., Almendros C. (2009). Short motif sequences determine the targets of the prokaryotic CRISPR defence system. Microbiology.

[23] Fineran P.C., Gerritzen M.J., Suarez-Diez M., Kunne T., Boekhorst J., van Hijum S.A., Staals R.H., Brouns S.J. (2014). Degenerate target sites mediate rapid primed CRISPR adaptation. Proc. Natl. Acad. Sci. U.S.A..

[24] Sternberg S.H., Redding S., Jinek M., Greene E.C., Doudna J.A. (2014). DNA interrogation by the CRISPR RNA-guided endonuclease Cas9. Nature.

[25] Anders C., Niewoehner O., Duerst A., Jinek M. (2014). Structural basis of PAM-dependent target DNA recognition by the Cas9 endonuclease. Nature.

[26] Hochstrasser M.L., Taylor D.W., Bhat P., Guegler C.K., Sternberg S.H., Nogales E., Doudna J.A. (2014). CasA mediates Cas3-catalyzed target degradation during CRISPR RNA-guided interference. Proc. Natl. Acad. Sci. U.S.A..

[27] Wiedenheft B., van Duijn E., Bultema J., Waghmare S., Zhou K., Barendregt A., Westphal W., Heck A., Boekema E., Dickman M. (2011). RNA-guided complex from a bacterial immune system enhances target recognition through seed sequence interactions. Proc. Natl. Acad. Sci. U.S.A..

[28] Lee D.G., Urbach J.M., Wu G., Liberati N.T., Feinbaum R.L., Miyata S., Diggins L.T., He J., Saucier M., Deziel E. (2006). Genomic analysis reveals that Pseudomonas aeruginosa virulence is combinatorial. Genome Biol..

[29] Cady K.C., Bondy-Denomy J., Heussler G.E., Davidson A.R., O'Toole G.A. (2012). The CRISPR/Cas adaptive immune system of Pseudomonas aeruginosa mediates resistance to naturally occurring and engineered phages. J. Bacteriol..

[30] Bondy-Denomy J., Pawluk A., Maxwell K.L., Davidson A.R. (2013). Bacteriophage genes that inactivate the CRISPR/Cas bacterial immune system. Nature.

[31] Wiedenheft B. (2013). In defense of phage: viral suppressors of CRISPR-mediated adaptive immunity in bacteria. RNA Biol..

[32] Bauer M., Metzler R. (2012). Generalized facilitated diffusion model for DNA-binding proteins with search and recognition states. Biophys. J..

[33] Hu L., Grosberg A.Y., Bruinsma R. (2008). Are DNA transcription factor proteins maxwellian demons. Biophys. J..

[34] Zhou H.X. (2011). Rapid search for specific sites on DNA through conformational switch of nonspecifically bound proteins. Proc. Natl. Acad. Sci. U.S.A..

[35] Slutsky M., Mirny L.A. (2004). Kinetics of protein–DNA interaction: facilitated target location in sequence-dependent potential. Biophys. J..

[36] Matos R.G., Barbas A., Arraiano C.M. (2010). Comparison of EMSA and SPR for the characterization of RNA–RNase II complexes. Protein J..

[37] Jackson R.N., Golden S.M., van Erp P.B., Carter J., Westra E.R., Brouns S.J., Van Der Oost J., Terwilliger T.C., Read R.J., Wiedenheft B. (2014). Crystal structure of the CRISPR RNA-guided surveillance complex from Escherichia coli. Science.

[38] Mulepati S., Heroux A., Bailey S. (2014). Crystal structure of a CRISPR RNA-guided surveillance complex bound to a ssDNA target. Science.

[39] Wiedenheft B., Lander G.C., Zhou K., Jore M.M., Brouns S.J.J., van der Oost J., Doudna J.A., Nogales E. (2011). Structures of the RNA-guided surveillance complex from a bacterial immune system. Nature.

[40] Zhao H., Sheng G., Wang J., Wang M., Bunkoczi G., Gong W., Wei Z., Wang Y. (2014). Crystal structure of the RNA-guided immune surveillance Cascade complex in Escherichia coli. Nature.

[41] Sinkunas T., Gasiunas G., Waghmare S.P., Dickman M.J., Barrangou R., Horvath P., Siksnys V. (2013). In vitro reconstitution of Cascade-mediated CRISPR immunity in Streptococcus thermophilus. EMBO J..

