# Supplementary material for: Mechanism of foreign DNA recognition by a CRISPR RNA-guided surveillance complex from Pseudomonas aeruginosa
Source: Nucleic Acids Res. 2015 Feb 8;43(4):2216–22. doi: 10.1093/nar/gkv094 (PMC4344526; doi:10.1093/nar/gkv094)
Supplement: SUPPLEMENTARY DATA [file supp_43_4_2216__index.html]

Mechanism of foreign DNA recognition by a CRISPR RNA-guided surveillance complex from Pseudomonas aeruginosa — Mechanism of foreign DNA recognition by a CRISPR RNA-guided surveillance complex from Pseudomonas aeruginosa — SUPPLEMENTARY DATA 

# Mechanism of foreign DNA recognition by a CRISPR RNA-guided surveillance complex from *Pseudomonas aeruginosa*

## SUPPLEMENTARY DATA

**Files in this Data Supplement:**

- SUPPLEMENTARY DATA
