# Supplementary material for: Mechanism of foreign DNA recognition by a CRISPR RNA-guided surveillance complex from Pseudomonas aeruginosa
Source: Nucleic Acids Res. 2015 Feb 8;43(4):2216–22. doi: 10.1093/nar/gkv094 (PMC4344526; doi:10.1093/nar/gkv094)
Supplement: SUPPLEMENTARY DATA [file supp_gkv094_nar-03567-h-2014-File003.zip › NAR-03567-H-2014_SOM.docx]

**Supplemental Material**

**Supplemental Table 1.** Equilibrium dissociation constants (K_D_) for ssDNA and dsDNA targets.

**Supplemental Table 2.** DNA oligonucleotides used in EMSAs, SPR, and competition assays. Protospacer sequences are shown in light blue and PAM or PAM mutations are shown in red.

**Supplemental Figure 1.** CRISPR systems have sub-Type-specific PAMs. Consensus PAMs are shown for CRISPR subtypes with well-characterized PAMs. **A.** In the Type I-E system from *Escherichia coli*, five different PAMs have been shown to elicit direct interference, with an additional 22 PAMs (not shown) that can direct primed spacer acquisition (20). **B.** Like *E. coli*, *Streptococcus thermophilus* also has a Type I-E system, but the PAM is more restricted. The consensus PAM is two consecutive thymine-adenine (A-T) base pairs, but only one A-T base pair is required. This requirement for an A-T base pair is also observed for the 5 PAMs that trigger direct interference by Cascade. **C.** Stringent target discrimination by Csy requires two sequential G-C base pairs. **D.** The Cas9 protein from *Streptococcus pyogenes* recognizes two consecutive C-G base pairs that are separated from the protospacer (blue) by a single base pair (N) that does not contribute to target detection (22,23).

**Supplemental Figure 2.** Chemical modification to guanines result in binding defects for targets with double-stranded PAMs. Representative EMSAs performed using a titration gradient (0, 0.001, 0.01, 0.05, 0.1, 0.5, 1, 10, 100, 1000nM) of the Csy complex, with either dsDNA or ssDNA targets containing wild type (GG) or chemically modified PAMs. The guanines were replaced with inosine (I), two amino purine (2AP), or 7 deaza guanine (7DG).

**Supplemental Figure 3.** Kinetic models for interaction between the Csy complex and a dsDNA target containing a PAM and a protospacer. **A)** Kinetic data for a concentration series of Csy complex (colored lines) were fit with a Langmuir binding model (black lines). For each concentration, the difference between the data and the curve fit (residuals) is plotted with gates indicating 5% (green lines) and 10% (red lines) of R_max_. A systematic pattern of residuals in excess of 10% of R_max_ indicates a poor curve fit. The Langmuir binding model does not fit the association or dissociation phases of these data, indicating that Csy complex target binding does not follow standard Langmuir binding kinetics. **B)** The same kinetic data were also fit with a two-state binding model (black lines). This model results in a better fit, with substantial deviations (residuals) limited mainly to the early association phase.

**Supplemental Figure 4.** Csy complex binds GC/GC dinucleotides. **A)** Results of competition assays with labeled dsDNA targets and unlabeled competitor dsDNA that was either GG-rich, or contained no GGs. Assays were performed with 5, 10, and 15 minutes incubations.
